# Instructing participants about the random assignment of patients to treated and non-treated conditions does not diminish causal illusions

**DOI:** 10.1098/rsos.251004

**Published:** 2025-11-12

**Authors:** Ainoa Barreiro, Javier Rodríguez-Ferreiro, Itxaso Barberia

**Affiliations:** ^1^ Grup de Recerca en Cognició i Llenguatge (GRECIL), Departament de Cognició, Desenvolupament i Psicologia de l’Educació, Secció Processos Cognitius, Institut de Neurociències (UBneuro), Universitat de Barcelona (UB), Barcelona, Spain

**Keywords:** causal illusion, alternative causes, contingency learning, instructions, causal learning

## Abstract

People sometimes perceive causal relationships between non-contingent events. When having to assess the contingency between a putative cause and an outcome, it is vital to ensure that all other causal forces are held constant whether the studied cause is present or not. Nevertheless, a recent work suggested that, in conventional contingency learning scenarios, people do not necessarily assume that it is the case. A possible contributing factor to this asset is that instructions in contingency learning tasks do not typically clarify this point. In two experiments, we manipulated the task instructions so that only half of the participants were explicitly informed that the introduction of the putative cause was randomly decided for each trial. The second experiment further instructed participants in the implications of random assignment regarding the control of alternative causes. Results of both experiments indicated that the manipulation of the instructions had no impact on the strength of causal illusions (minimum *BF*_*01*_ = 5.853). Nevertheless, the susceptibility to develop causal illusions was related to a lack of an appropriate consideration of alternative causal forces and a tendency to overweight the importance of the probability of the outcome in the presence, rather than in the absence, of the putative cause.

## 1. Introduction

Humans have an inherent capacity to perceive cause–effect relationships in natural environments, which can be observed even in the early stages of infancy [1,2]. From an evolutionary perspective, the importance of perceiving causality seems evident. Our ancestors might have had to determine the presence of a predator based on the trails observed in the path or judge the poisonous potential of a given plant. Despite no longer living in these environments, in modern societies, people still face situations that require causal inferences, such as judging the efficacy of hair products they saw to increase hair growth or testing whether changing certain diet habits can help them reduce weight.

To assess whether a causal relationship exists between two events, one must evaluate whether the putative cause and outcome covary. In other words, consider occasions in which (i) the putative cause and effect were present, (ii) the putative cause was present but the outcome was not, (iii) the putative cause was absent and the outcome was present, and (iv) both the putative cause and outcome were absent.

For binary cues and outcomes, these four combinations of events can be easily differentiated, and causal judgements can be studied by manipulating their frequency in a contingency task (though, see [3,4] for continuous variables). Typically, in these tasks, participants are required to evaluate the causal relationship between two events, such as taking a drug (putative cause) and recovering from an illness (outcome) based on information from alleged patients presented trial-by-trial. In each trial, the participant is provided with information about whether that patient took the drug or not, and whether or not the patient recovered. At the end of the trials, the participant must assess to what extent they believe there is an association between drug intake and recovery (i.e. to provide a causal judgement).

It is widely accepted that Δ*P* [5] can be an accurate descriptor of contingency. The index is computed by comparing the probability of the outcome occurring in the presence and absence of the (putative) cause, *P*(*O*|*C*) and *P*(*O*|*~C*), respectively. Studies suggest people are capable of making accurate causal judgements [6–8]. Nonetheless, reports have also detected some systematic biases in these judgements, such as the illusion of causality or causal illusion (e.g. [9–12]) which occurs when people perceive a causal relationship between non-contingent events (i.e. *P*(*O*|*C*) = *P*(*O~C*), and therefore Δ*P* = 0). These illusions have been linked to social challenges of major concern, such as the endorsement of unwarranted beliefs [13–15] and fake news discriminability [16].

In a recent work, Lovibond *et al.* [17] proposed an explanation for the observation of causal illusions. They suggested participants might not share the researchers’ assumption regarding the presence of the same alternative causes (i.e. hidden factors that might also be effective generators of the outcome) whether the candidate cause is present or not. For example, following the aforementioned health-related causal learning scenario, some participants will typically recover after taking the key drug, but recovery will also eventually be observed in some of the patients who did not take the drug. To explain the existence of patients healing without the drug, other reasons for recovery should be presumed (e.g. natural recovery, physiological factors), even if they have not been made explicit by the experimenters. In this sense, for Δ*P* to be considered an accurate descriptor of the specific impact of the candidate cause over the probability of the outcome, one needs to accept that these alternative causes (e.g. having a strong immune system, age, pre-existing health conditions) remain constant irrespective of the presence or absence of the candidate cause.

However, as demonstrated by Lovibond *et al.* [17], participants do not unvaryingly assume unknown alternative causes to be equally operating in both treated and untreated patients who recovered. After completing a contingency-learning task similar to the one described above (see Lovibond *et al.* [17], Experiment 2), their participants indicated, through a forced-choice question, which was the reason for recovery in those patients not taking the treatment. Participants answered this question (*alternative-causes* test) by mentioning alternative hidden causes of recovery (e.g. spontaneous recovery, immunity…). Next, they had to indicate what they thought was the reason for recovery among those patients who did take the treatment. Interestingly, those participants who answered that recovery in these later patients was mainly due to the same aspect they had indicated for patients recovering without the drug showed less intense causal illusions (i.e. lower causal ratings) than those attributing recovery mainly or partly to the treatment. From their results, the authors concluded that different participants might diverge in their assumptions regarding the permanence of alternative causes when the candidate cause is present and, crucially, that all these interpretations might be considered normative. In consequence, causal illusions might not be ‘illusions’ after all.

Admittedly, researchers investigating the development of causal illusions in the laboratory might have unfairly concluded that participants should assume the permanence of the alternative causes of the outcome. As noted by Lovibond *et al.* [17], contingency-learning tasks involving medical scenarios generally include quite vague instructions that do not have any indication as to what criteria determined who took and who did not take the drug (e.g. [3,11,18,19]). Thus, participants might have plausible explanations on why some patients were given the treatment and some were not. For instance, they might presume that only those patients with severe symptoms were administered the treatment. In experimental research, random assignment of patients is ideal to optimize the presence of the same alternative causes in those who take the drug and those who do not, and therefore, the gold standard when evaluating the effectiveness of a real treatment.

The goal of Experiment 1 was to evaluate if, when hinting participants regarding the equal potential influence of hidden alternative causes both in patients who took and who did not take the drug, the intensity of causal illusions would be diminished. For this, volunteers were randomly assigned to one of two conditions. One of the conditions (*Control*) was analogous to that of previous studies, in which no hint was given regarding the decision to administer the drug. In the second condition (*Random*), participants were given indications that, for each patient, whether the experimental drug was given was decided randomly.

If Lovibond *et al.*’s suggestion was correct, we expected a reduction in causal illusions (i.e. causal ratings) in the *Random* condition compared with the *Control* one (hypothesis 1), because the proportion of participants disregarding alternative causes for explaining the occurrence of recovery in patients that took the drug in the *alternative-causes* test would be lower in the former condition compared with the second one (hypothesis 2).

Moreover, if our experimental manipulation was effective, then we would expect participants in the two conditions to differentially perceive the relevance of information regarding healings produced in the presence and absence of the putative cause. Extending Lovibond *et al*.’s procedure, we explicitly asked our participants how important they considered the probability of recovery when the cause is present (i.e. *P*(*O|C*)) and absent (i.e. *P*(*O|~C*)) for causal estimation. We expected that the difference between them would be lower in the *Random* condition than in the *Control* condition (hypothesis 3).

Regarding the relationship between the different dependent variables, we expected that the alternative chosen in the *alternative-causes* test would predict differences in the numerical causal rating (hypothesis 4) as found in Lovibond *et al.* [17], and that there would be a positive correlation between the *P*(*O|C*) vs. *P*(*O|~C*) *importance* value and causal ratings (hypothesis 5).

## 2. Experiment 1

### 2.1. Method

#### 2.1.1. Participants

We recruited a sample of 260 participants through Prolific (130 in each condition) to achieve a power of 0.80 in an independent samples *t*‐test for a small-to-medium effect size (*d* = 0.35). The study protocols (for this and the next experiment) were approved by the ethics committee of the University of Barcelona, and the participants gave informed consent before their participation in the study. The study was pre-registered in AsPredicted (https://aspredicted.org/g9jw-vbsh.pdf).

#### 2.1.2. Design and procedure

Participants were randomly assigned to *Random* or *Control* conditions. In the *Random* condition, initial instructions (see the Supplementary material) indicated that patients would be randomly assigned to experimental (drug) and control (no-drug) groups by a coin-tossing system. The *Control* condition received similar instructions but without any reference to the random assignment of patients to drug vs. no-drug groups.

To further reinforce the idea of random assignment in the *Random* condition, these participants could observe, trial by trial, a coin tossing which determined whether each subsequent patient received the drug or not. On each occasion, they could read ‘A new patient has a headache and, since the coin turned up heads/tails, the experimental drug is administered/NOT administered’, together with the corresponding picture of the experimental drug or the same drug crossed out, an animation of a coin being tossed and turning heads or tails, and a schematic person with a headache. In the *Control* condition, the participants received the same information, except that no mention of the random assignment or coin tossing was made.

For each new fictitious patient, participants from both conditions had to answer the yes or no question ‘Will the patient recover from the headache in the following two hours?’. After their choice, they were presented with the text ‘The patient recovered from the headache’ or ‘The patient did NOT recover from the headache’, depending on whether the outcome was meant to occur or not in that specific trial, and the schematic image of the patient turned into a recovered state or remained sick, respectively.

All participants were exposed to a total of 48 patients. Half of them received the experimental drug, and the other half did not receive any treatment. Moreover, three-quarters of patients who took the experimental drug (18 out of 24) experienced relief from the headache, and also three-quarters of patients who did not take any treatment (18 out of 24) experienced such relief. Therefore, the contingency between the experimental drug and the relief from headaches was null (Δ*P* = 0).

After finishing the learning phase, participants were exposed to three different tests. First, they provided conventional *causal ratings*, in which they had to indicate the causal influence of the drug on the recovery. This causal question was answered on a scale from 0 to 100 (i.e. ‘To what extent do you think that the experimental drug is effective against headaches? A value of 0 indicates that the drug is not at all effective against headaches, while a value of 100 indicates that the drug is totally effective as a treatment for headaches. Intermediate values indicate intermediate effectiveness of the drug. You can enter any value between 0 and 100’.). Also, for exploratory reasons, once the participants gave their numerical response, they were asked to explain, through an open question [20], their reasons for it (i.e. ‘You just gave a response of __ when asked to indicate which was the effectiveness of the experimental drug in a scale from 0 (not effective at all) to 100 (totally effective). Could you explain what information you took into account and what reasoning you followed to reach that conclusion?’).

Second, we introduced a forced-choice test (*alternative-causes* test) slightly adapted from Lovibond *et al.* [17] with two questions. First (*cell-C question*), participants read ‘Some patients who did not take the experimental drug recovered from their headaches. Did you think this was…’ and had the following response options: (i) ‘because of the natural course of the illness (spontaneous recovery)’, (ii) ‘because these patients had stronger immune systems’, (iii) ‘because of something else (please describe below)’, (iv) ‘I didn’t think about this issue during the experiment’. The second question (*cell-A question*) read ‘Some patients who took the experimental drug recovered from their headaches. Did you think this was…’ and had the following response options: (i) ‘because of the experimental drug’,^1^ (ii) ‘because of the same reason you chose in the previous question’, (iii) ‘both because of the experimental drug and because of the same reason you chose in the previous question’, (iv) ‘I didn’t think about this issue during the experiment’.

Finally, participants answered two questions (*P*(*O|C*) vs. *P*(*O|~C*) *importance* test) in a 0 (not important at all) to 100 (extremely important) scale, regarding the importance of *P*(*O*|*C*) and *P*(*O*|~*C*) in order to evaluate the effectiveness of the experimental drug, which were, respectively: (i) ‘In order to evaluate the effectiveness of the experimental drug, to what extent do you consider that it is relevant to have information regarding the percentage of patients who recovered from headaches, among those who took the experimental drug?’, and (ii) ‘In order to evaluate the effectiveness of the experimental drug, to what extent do you consider that it is relevant to have information regarding the percentage of patients who recovered from headaches, among those who did NOT take the experimental drug?’. In both cases, it was explained to them that ‘A value of 0 indicates that the information is not important at all and a value of 100 indicates that it is extremely important. Intermediate values indicate moderate relevance. You can enter any value between 0 and 100’.

The order of these three tests was the same for all participants (first, *causal ratings*, second *alternative-causes* test, and third *P*(*O|C*) vs. *P*(*O|~C*) *importance* test), but the order in which the two questions were asked in the *P*(*O|C*) vs. *P*(*O|~C*) *importance* test was randomly decided for each participant.

### 2. Results

2. 

We used JASP (version 0.18.3) to conduct the statistical analyses.^2^ The datasets from which the analyses of this and the next experiment have been performed can be found at OSF (https://osf.io/tzp7c/overview).

The intensity of causal ratings (i.e. causal illusions) was similar in both *Random* and *Control* conditions (*mean*_Control_ = 52.546, s.d. = 26.985; *mean*_Random_ = 54.508, s.d. = 25.345), *t*(258) = −0.604, *p* = 0.546, *d* = −0.075, *BF_01_* = 6.181. Therefore, contrary to our hypothesis 1, instructing participants about the random assignment of patients to the treated or non-treated group did not affect numerical causal estimations. Indeed, the obtained Bayes factor indicates that there is moderate evidence supporting this null hypothesis.

Regarding the *cell-C question* of the *alternative-causes* test, in which participants had to determine the reasons for recovery in patients not taking the drug, most of the participants (88%) indicated that this happened due to spontaneous recovery. Of more interest was the pattern of responses regarding the *cell-A question* of this test, in which participants had to indicate the reasons for the recovery of those patients who received the drug, which we hypothesized would be dependent on the participants belonging to the *Random* or *Control* condition. Table 1 shows the distribution of responses to this question. Contrary to hypothesis 2, responses turned out to be unaffected by the experimental manipulation,^3^ *X*^2^ (2, 255) = 1.002, *p* = 0.606, *BF_01_* = 20.782. As can be seen in the table, the most frequent response was attributing the recovery to both the drug and the alternative cause mentioned in the first question (58%), followed by attributing the recovery solely to this alternative cause (24%) and attributing the recovery solely to the drug (16%).

**Table 1.** Response distributions of the explanations given for Cell A for each instruction condition in Experiment 1.

	explanations for Cell A			
instructions	drug	alternative	drug and alternative	have not thought
control	22	34	73	1
random	19	28	79	4
**total**	41 (16%)	62 (24%)	152 (58%)	5 (2%)

Next, we analysed whether participants in the *Random* condition showed a lower difference in the importance attributed to the *P*(*O*|*C*) relative to the *P*(*O*|~*C*) (*P(O|C*) vs. *P*(*O|~C*) *importance* test), as stated in our hypothesis 3. Contrary to our hypothesis and consistent with our results above, there were no significant differences between conditions in this relative importance measure (*mean*_Control_ = 5.469, s.d. = 20.589; *mean*_Random_ = 3.885, s.d. = 18.933), *t*(258) = 0.646, *p* = 0.519, *d* = 0.080, *BF_01_* = 6.031. Although this analysis was not pre-registered, it is worth noting that the *P*(*O*|*C*) was considered generally more important than *P*(*O*|~*C*), a one-sample *t*‐test of the difference between these two values against a theoretical value of zero being significant, *t*(259) = 3.817, *p* < 0.001, *d* = 0.237, *BF_10_* = 77.107.

**Figure 1.**
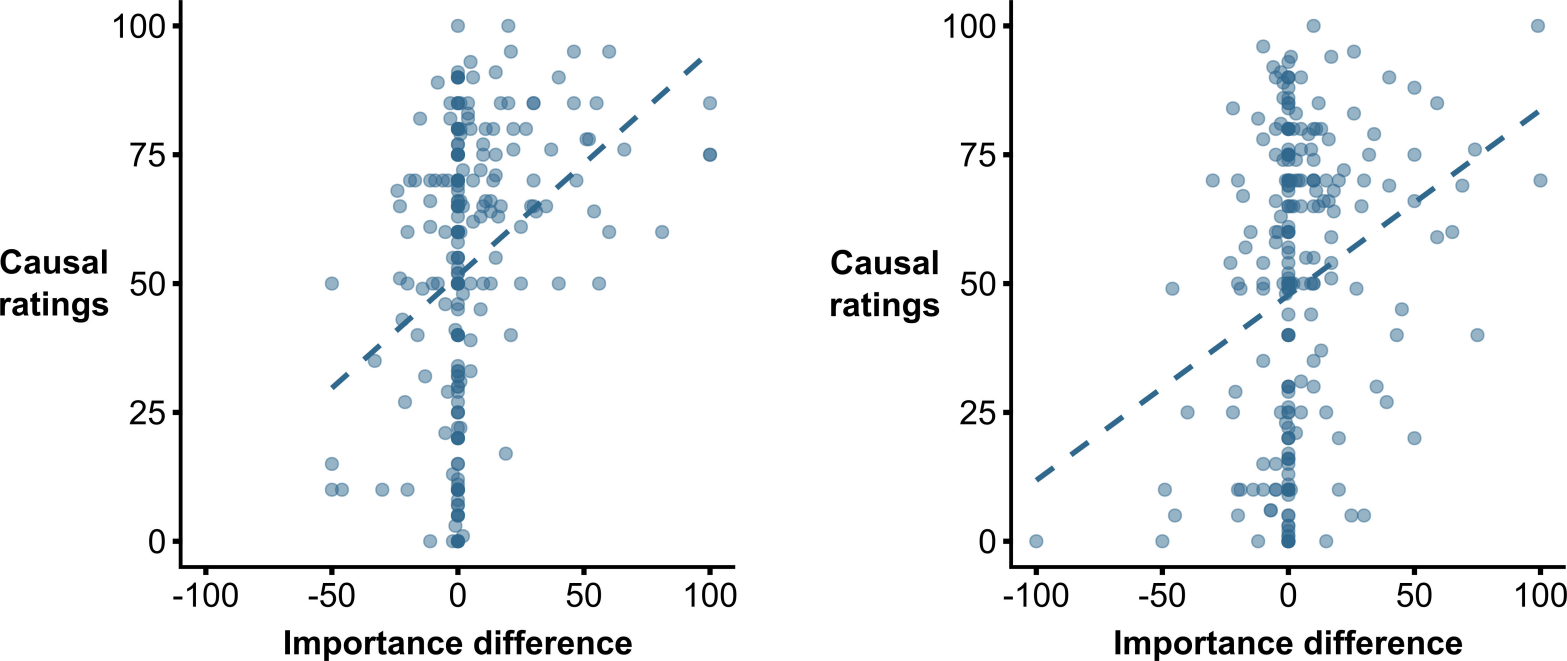
Association between causal ratings and scores in the *P*(*O*|*C*) vs. *P*(*O*|~*C*) *importance* test in Experiment 1 (left panel) and Experiment 2 (right panel).

We then analysed the relationship between the different dependent variables of the study. First (hypothesis 5), as shown in figure 1 (left panel), we observed that those participants with a higher value in the *P*(*O|C*) vs. *P*(*O|~C*) *importance* test also reported higher causal ratings, *r* = 0.329, *p* < 0.001, *BF_10_* = 185 178.162.

**Figure 2.**
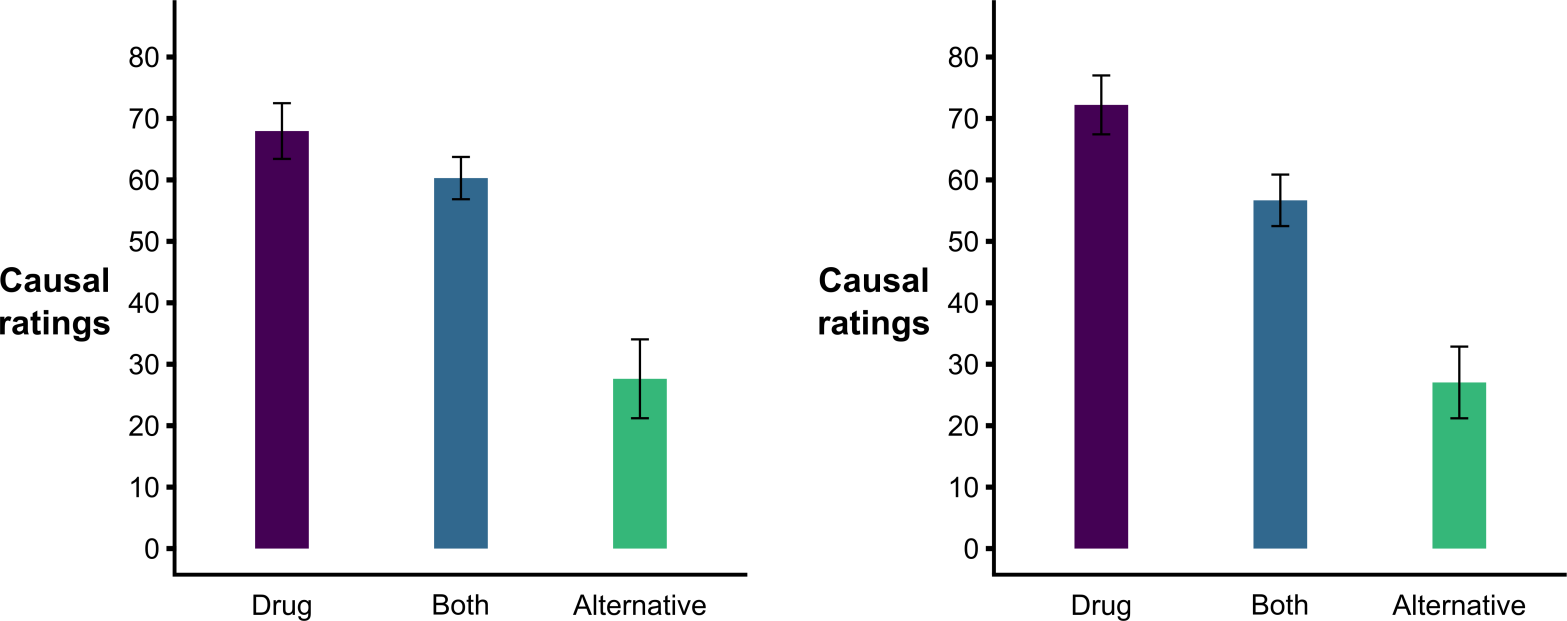
Bar graphs of the observed causal ratings based on the *alternative-causes* test response in Experiment 1 (left panel) and Experiment 2 (right panel). Note: bars depict the mean causal ratings for each response in the *alternative-cause* test, and the error bars define the lower and upper bounds of the confidence interval.

Second (hypothesis 4) and analogous to Lovibond *et al*.’s [17] analysis, an ANOVA with the answers to the *cell-A question* of the *alternative-causes* test as the factor (with levels: ‘drug’, ‘alternative’, or ‘both’, corresponding to the three first options of response) and causal ratings as the dependent variable revealed a significant effect, *F*(2, 252) = 61.561, *p* < 0.001, *η_p_*^2^ = 0.328. Post hoc Tukey tests revealed that those participants answering that an alternative cause other than the drug was responsible for recovery in patients who took the drug developed less intense causal illusions than those answering either that the drug was responsible for this recovery, *p* < 0.001, or that both the drug and the alternative cause were responsible, *p* < 0.001. These later two groups did not differ in their causal ratings, *p* = 0.109 (see figure 2, left panel).

**Figure 3.**
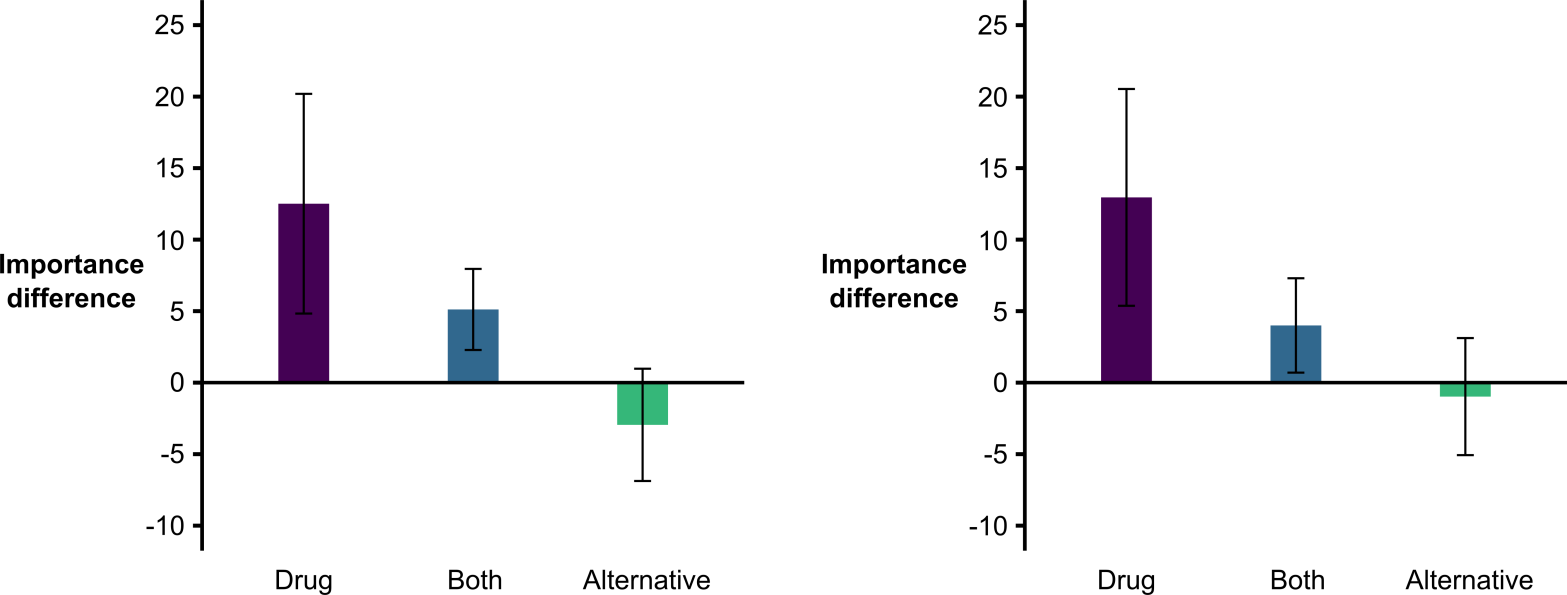
Bar graphs of the scores in the *P*(*O*|*C*) vs. *P*(*O*|~*C*) *importance* test based on the *alternative-causes* test response in Experiment 1 (left panel) and in Experiment 2 (right panel). Note: bars depict the mean importance difference for each response in the *alternative-cause* test, and the error bars define the lower and upper bounds of the confidence interval.

Finally, although not pre-registered, and following the suggestion of a reviewer, we decided to analyse the association between the *alternative-causes* and the *P*(*O|C*) vs. *P*(*O|~C*) *importance* tests (see figure 3, left panel). To do so, we ran an ANOVA on relative importance ratings obtained in the latter test with the answers to the *cell-A question* of the *alternative-causes* test as the factor (with levels: ‘drug’, ‘alternative’ or ‘both’, *mean*_Drug_ = 12.512, s.d. = 24.336; *mean*_Both_ = 5.118, s.d. = 17.689, *mean*_Alternative_ = −2.952, s.d. = 15.460). The analysis returned a significant effect, *F*(2, 252) = 9.027, *p* < 0.001, *η_p_*^2^ = 0.067, and subsequent post hoc Tukey tests were consistent with previously found effects, as those participants answering ‘alternative’ in the *alternative-causes* test obtained lower relative importance ratings than those answering either ‘both’ or ‘drug’ (*p* = 0.011 and *p* < 0.001, respectively), while these latter groups did not significantly differ, *p* = 0.060.

## 3. Experiment 2

In Experiment 1, we found that the task instruction manipulation did not impact any of our dependent variables. However, a potential limitation was that, while our manipulation consisted of informing that the administration of the drug was randomly decided for each patient, we did not include any questions to verify that participants actually understood the implications of a random assignment. Therefore, in a second experiment, we further instructed participants in the *Random* condition on the rationale of random assignment, and we introduced an initial manipulation check in order to confirm that they had understood our instructions. Those participants who did not pass the manipulation check were not allowed to proceed to the contingency learning task.

### 3.1. Method

#### 3.1.1. Participants

In order to achieve the same power as in Experiment 1, we recruited a sample of 260 valid participants through Prolific (131 and 129 participants in *Control* and *Random* conditions, respectively). In *Random* condition, 26 participants did not pass our initial manipulation check (see below) and, therefore, were not allowed to complete the contingency learning task. The study was pre-registered in AsPredicted (https://aspredicted.org/8t8w-f4vs.pdf).

#### 3.1.2. Design and procedure

Participants were randomly assigned to *Random* or *Control* conditions. The procedure was identical to Experiment 1, except that now participants in the *Random* condition were not only informed that a coin-tossing system would be used to assign patients to either the treated or non-treated groups, but further told that ‘random assignment of the patients to the treated and non-treated groups is common-practice when evaluating the effectiveness of an experimental drug, because it promotes that aspects that might influence recovery other than the drug itself are equally distributed among the two groups of patients’. Moreover, they were asked to subsequently answer the multiple-choice question ‘What is the goal of random assignment of patients to treated and untreated groups when evaluating the effectiveness of an experimental drug?’, with the correct answer being ‘to ensure that other possible sources of recovery are maintained constant among the two groups’ (see the Supplementary material for the complete instructions and manipulation-check question).

The only other difference in Experiment 2, which otherwise was equal to Experiment 1, was that now only half of the participants (*n* = 130) were first asked to provide their causal rating, and then answered the *alternative-causes* test and *P*(*O|C*) vs. *P*(*O|~C*) *importance* test, whereas in the rest of the participants the causal question was presented after both the *alternative-causes* test and *P*(*O|C*) vs. *P*(*O|~C*) *importance* test had been completed.

### 3.2. Results

The results observed in Experiment 2 were largely consistent with those obtained in Experiment 1. First, Random and Control conditions did not differ in their causal ratings *mean*_Control_ = 47.969, s.d. = 29.576; *mean*_Random_ = 50.465, s.d. = 28.458), *t*(258) = −0.693, *p* = 0.489, *d* = −0.086, *BF_01_* = 5.853.

Second, as observed in Experiment 1, most of the participants (87%) indicated that recovery in non-treated patients was due to spontaneous recovery in their answers to the first question of the *alternative-causes* test. Crucially, replicating our previous result, their answers to the second question of the same test (table 2) were independent of the (*Random* vs. *Control*) condition,^4^ *X*^2^ (2, 254) = 0.935, *p* = 0.627, *BF_01_* = 106.625. The most prevalent response was again considering that recovery happened both because of the drug and the alternative cause mentioned before (50%), and the least prevalent answer attributed recovery exclusively to the drug (17%).

**Table 2.** Response distributions of the explanations given for Cell A for each instruction condition in Experiment 2*.*

	explanations for Cell A			
instructions	drug	alternative	drug and alternative	have not thought
control	20	45	64	2
random	23	37	65	4
**total**	43 (17%)	82 (32%)	129 (50%)	6 (2%)

Third, the results of the *P*(*O|C*) vs. *P*(*O|~C*) *importance* test were again similar for *Random* and *Control* conditions (*mean*_Control_ = 5.595, s.d. = 20.791; *mean*_Random_ = 2.496, s.d. = 19.571), *t*(258) = 1.237, *p* = 0.217, *d* = 0.153, *BF_01_* = 3.559. Moreover, although not pre-registered and secondary for our goals, the *P*(*O*|*C*) was again assumed to be more relevant than *P*(*O*|~*C*), *t*(259) = 3.237, *p* = 0.001, *d* = 0.201, *BF_10_* = 11.157. Furthermore, when analysing the relationship between answers to this test and causal ratings (see figure 1, right panel), we replicated the previously observed positive relationship, *r* = 0.250, *p* < 0.001, *BF_10_* = 309.737.

Fourth (see figure 2, right panel) and analogous to Lovibond *et al*.’s [17] analysis, we again observed that answers to the second question of the *alternative-causes* test (i.e. reasons for recovery in treated patients, including the levels ‘drug’, ‘alternative’, or ‘both’) predicted differences in causal ratings, *F*(2, 251) = 62.270, *p* < 0.001, *η_p_^2^* = 0.332. Note that post hoc Tukey tests revealed differences between all three levels (‘drug’ > ‘both’ > ‘alternative’, all comparisons *p* < 0.001), while in Experiment 1 the difference between ‘drug’ and ‘both’ levels did not reach significance (*p* = 0.109).

Finally, an analogous ANOVA to that run in Experiment 1 with answers to the *cell-A question* as the factor and importance ratings as the dependent variable (*mean*_Drug_ = 12.953, s.d. = 24.632; *mean*_Both_ = 4.000, s.d. = 18.943, *mean*_Alternative_ = -0.951, s.d. = 18.629) indicated a consistent significant effect, *F*(2, 251) = 6.879, *p* = 0.001, *η_p_^2^* = 0.052. In this case, although the means were ordered in a similar way as that observed in Experiment 1 (see figure 3, right panel), the difference between ‘drug’ and either ‘both’ (*p* = 0.030) or ‘alternative’ (*p* < 0.001) levels reached significance, while ‘both’ and ‘alternative’ levels did not differ, *p* = 0.185.

## 4. Discussion

In a recent paper, Lovibond *et al.* [17] highlighted that the instructions in contingency tasks are usually unspecific regarding whether the presence of the same alternative causes should be assumed in both cause-present and cause-absent trials. They argued that this ambiguity might explain individual differences in the explanations for cause-present outcomes and, consequently, affect the resulting causal judgements.

In our research, we manipulated the task instructions so that they would explicitly state that the decision to administer the putative cause (the drug) was random (i.e. toss of a coin), therefore, promoting the key idea of the presence of the same hidden factors. The analyses of the answers given to the *alternative-causes* test corroborated that participants diverged in their assumptions about the reasons for the recovery of those patients who had received the drug. Nevertheless, we did not find any difference in the responses to this test between participants in the *Random* and *Control* conditions. Thus, the fact that individuals do not always assume independence between the presence of the putative cause and the presence of alternative causes cannot be attributed to the lack of specific instructions, at least in regard to information about the random assignment of patients to treated and untreated groups. In fact, differences between *Random* and *Control* conditions did not emerge whether participants in the *Random* condition were only informed about the random assignment of patients to treated and untreated groups (Experiment 1) nor whether they were further instructed about the implications of such random assignment and were tested to confirm that they had comprehended our explanation (Experiment 2). Our manipulation was consistently ineffective in altering the strength of causal illusions, the perceived importance of *P*(*O|C*) and *P*(*O|~C*) to assess the drug efficacy, or the reasons for recovery of treated patients given in the *alternative-cause* test.

It is worth noting that, although our experimental manipulation did not affect any of our dependent variables, all these dependent variables appeared to be associated. First, we found a link between the answers to the *alternative-cause* test and the causal ratings given by participants. More specifically, participants who believed the recovery of patients who received the drug was due to an alternative cause (e.g. a stronger immune system, natural recovery) developed weaker causal illusions than those who attributed the recovery uniquely to the drug. Causal illusions of those participants who attributed recovery of treated patients both to the drug and the alternative cause (i.e. answered ‘both’) fell somewhere in between the previous two groups. Namely, they significantly differed both from those who attributed recovery solely to the drug and those who attributed recovery solely to the alternative in Experiment 2 (i.e. drug > both > alternative), while only differing from the latter group in Experiment 1 (i.e. (drug = both) > alternative). Taken together, we can conclude that partial or total attribution of recovery to the drug is associated with stronger causal illusions, while full attribution of recovery to alternatives is most clearly associated with decreased causal illusions. These findings replicate the pattern observed by Lovibond *et al.* [17] and suggest that the lack of appropriate consideration of alternative causal forces might be a key element in predicting susceptibility to causal illusions.

Our study also indicates that instructing participants about the random assignment strategy for promoting the presence of the same alternative causes does not improve such consideration of alternatives. However, other approaches focusing on alternative causes have improved causal inferences more effectively. For instance, Vadillo *et al.* [21] demonstrated that illusions of causality can diminish in the presence of alternative causes that can account for the outcomes observed. The prompt for their contingency task was to try to turn off the flashes on the computer screen by using a joystick, although in reality, there was no actual contingency between them. Furthermore, participants were alerted that if asterisks appeared (i.e. alternative cause) during the task, flashes would turn off immediately. Crucially, the presence of the asterisks was manipulated, so only half of the sample visualized them. Afterwards, participants had to rate both the influence of the asterisk and the joystick on the termination of the flashes. Interestingly, the results of the effectiveness ratings of the joystick to terminate the flashes indicated that those participants who had been exposed to the asterisks gave lower ratings than those who had not been exposed to them. Thus, they had developed weaker causal illusions.

In line with these findings, MacFarlane *et al.* [22] also examined the impact of alternative explanations on causal judgements. Participants received varying information about a multivitamin’s effectiveness and were asked to bid the amount of money they would be willing to pay for the product. The authors presupposed that the strength of the causal illusions developed would be reflected in the participant’s willingness to pay, that is, participants who had stronger illusions would bid a larger amount of money. Results indicated that those individuals who were provided with contingency information along with an explanation referring to possible alternative causes for the recovery (i.e. the fact that ‘modern diets provide ample vitamins for the body to maintain healthy function’) were less willing to pay than those who were only given general information about the product. Therefore, the data suggested that providing hidden factors that can account for the observed outcomes can help mitigate causal illusions.

Altogether, the studies by Vadillo *et al.* [21] and MacFarlane *et al.* [22] suggest that alternative causes are considered when they are either explicitly presented during training (i.e. asterisks in Vadillo *et al.* [21]) or they provide a specific plausible alternative explanation for the outcome (i.e. indications of well-balanced diets already containing the nutrients needed for optimal health functioning in MacFarlane *et al.* [22]). These two features might explain why our manipulation was unable to divert attention to alternative causes. In fact, the significance of explicitness and plausibility of alternative explanations has also been addressed in relation to a different phenomenon in causal learning (i.e. protection from extinction) [23].

In light of this, encouraging the appraisal of alternative causes could be a successful strategy to debias causal illusions. Considering the endorsement of pseudoscience has been linked to a susceptibility to causal illusions [14,15], this broadens the scope for developing new interventions. This approach has already been taken to target the detrimental effects of misinformation [24–26] and to improve Bayesian reasoning [27], both showing encouraging results.

Moreover, our experimental design included an additional test (not present in the original study by Lovibond *et al.* [17], as an alternative way of evaluating the relevance given by people to alternative causes when evaluating a target cause, the *P*(*O|C*) vs. *P*(*O|~C*) *importance* test. Significantly, this measurement was linked to the responses given to the *alternative-causes* test. Participants who believed that the recovery of patients who received the drug was due to an alternative cause were less likely to weigh the relative importance of the *P*(*O*|*C*) as greater than that of the *P*(*O*|~*C*). These findings suggest that being unaware that the alternative causes explaining the recovery in drug-absent trials can also account for drug-present recoveries might foster overlooking why it is important to consider *P*(*O*|~*C*) information. That is, if one does not assume that the presence of alternative causes is the same in treated and untreated patients, then the sample of untreated patients constitutes a suboptimal comparison group for evaluating the drug, which might explain why its importance is undervalued.

In this sense, a person who understands the relevance of considering *P*(*O*|~*C*) in order to evaluate the real influence of a potential target cause should be at a lower risk of developing a causal illusion regarding the target cause. Our study indicated that stronger causal illusions correlate with a stronger importance attributed to *P*(*O*|*C*) relative to *P*(*O*|~*C*). This finding is consistent with a previous demonstration [28] that those participants with more intense and persistent causal illusions provide causal ratings that are more intensely associated with their *P*(*O*|*C*) estimations, compared with those participants with less intense and less durable causal illusions, whose causal ratings are, in contrast, more associated with their *P*(*O*|~*C*) estimations. While these previous observations suggest the potential implication of the subjective importance attributed to each of these conditional probabilities for understanding the intensity and persistence of causal illusions, in the present study, we introduced a direct measure of attributed importance by asking participants to explicitly rate the importance of each probability for causal inference.

Our results regarding the *P(O|C)* vs*. P(O|~C) importance* test are also somewhat linked to reports on active contingency learning tasks. The premise of these tasks is to assess the strength of a causal relationship between two events. Nonetheless, in each trial, the participant can decide whether the putative cause is administered. Based on this, Blanco *et al.* [29] and Torres *et al.* [15] illustrated that stronger causal illusions correlate with a tendency to frequently administer the cause. Hence, this behavioural tendency might indicate that individuals presume cause-present trials are highly relevant for determining cause–effect relationships. Indeed, as attested by Kao and Wasserman [30] and Wasserman *et al.* [31], when assessing contingency, people tend to primarily focus on cause-present trials and dismiss the events in which the cause is absent. Altogether, the current evidence suggests that causal illusions might be partially attributed to an asymmetry in the perceived importance of *P*(*O*|*C*) and *P*(*O~C*). Indeed, those participants who in our study were less likely to overweight *P*(*O*|*C*) relative to *P*(*O*|*~C*) developed weaker causal illusions. In line with this idea, educational interventions tackling causal illusions have focused on educating participants on the relevance of *P*(*O*|*~C*) when assessing a causal link [32,33].

Our present work is not without limitations. First, note that our goal was to evaluate whether a possible explanation for observations of causal illusions in zero contingency tasks is that participants assume that the presence of alternative causes vary across conditions in which the target cause is present or absent (i.e. treated and untreated patients). However, even if participants assume (or are led to assume) that these alternatives do not vary across conditions, they might still think that causal forces interact. That is, in the present scenario, a person might acknowledge that other aspects could facilitate recovery (e.g. a stronger immune system), but that those just boosted the influence of the drug, instead of being able to produce recovery by themselves. Future studies might investigate the extent to which participants’ assumptions about the independent or interactive influence of the target and alternative causal forces over the outcome affect their level of causal illusions.

Furthermore, note that the observed significant effects involve associations between the different dependent variables (causal ratings, explanations for recovery in treated patients, relative importance of conditional probabilities) and that our experimental manipulation did not have any significant impact on any of these variables. In this respect, even though we have speculated that poor consideration of alternative causes could facilitate causal illusions, it could also be the case that the already formed causal illusions yield the generation of explanations that are consistent with them. This could promote participants who believe in the drug’s effectiveness to subsequently attribute recovery of treated patients to the drug and/or to indicate that the proportion of recovery among untreated patients is relatively less important. Moreover, it could also be the case that all these variables are affected by third unconsidered factors. With regard to this, at least in the case of the relation between relative importance of conditional probabilities and causal illusions, future studies could try to determine whether the association also emerges when the *P*(*O|C*) vs. *P*(*O|~C*) *importance* test is completed before being exposed to any training, which would provide an unbiased measure of the general relevance attributed to these different pieces of information for causal inference.

In summary, we have provided evidence suggesting that the lack of consideration of alternative causal forces might be a contributing factor to the development of causal illusions. Nonetheless, our results suggest that this underestimation is not due to insufficient information given to participants.

## Data Availability

We pre-registered in AsPredicted Experiment 1 (https://aspredicted.org/g9jw-vbsh.pdf) and Experiment 2 (https://aspredicted.org/8t8w-f4vs.pdf). Data for both studies can be found in OSF (https://osf.io/tzp7c/overview). Supplementary material is available online [34].

## Footnotes

^1^
Note that in the original test by Lovibond *et al*. [17] alternatives ‘a’ and ‘b’ to this second forced choice question indicated ‘mostly because of the medicine’ and ‘mostly for the same reason you chose for the previous question’, respectively, but we avoided the ‘mostly’ expression in order to more clearly identify those patients that did consider alternative causes fully responsible or not responsible at all of generating the outcome, leaving alternative ‘c’ as the expected option for all those participants assuming that both causes might have contributed to some extent to the outcome.

^2^
Note that, for this and the next experiment, even though data violated the normality assumption, the analogous non-parametric tests were also conducted, and conclusions did not differ from those exposed.

^3^
Note participants who answered ‘I did not think about this issue during the experiment’ were removed from the statistical analysis. Yet, we also performed the analysis including these responses and the results did not vary.

^4^
As in Experiment 1, we excluded from the analysis those participants answering ‘I did not think about this issue during the experiment’, but the conclusions were the same when including these participants.

## Authors’ Notes

Informed consent was obtained from all individual participants included in the study.
